# Improvement of *Dscam* homophilic binding affinity throughout *Drosophila* evolution

**DOI:** 10.1186/s12862-014-0186-z

**Published:** 2014-08-27

**Authors:** Guang-Zhong Wang, Simone Marini, Xinyun Ma, Qiang Yang, Xuegong Zhang, Yan Zhu

**Affiliations:** State Key Laboratory of Brain and Cognitive Science, Institute of Biophysics, Chinese Academy of Sciences, 15 Datun Road, Beijing, 100101 China; Bioinformatics Division, TNLIST/Department of Automation, Tsinghua University, Beijing, 100084 China; Department of Computer Science and Engineering, Hong Kong University of Science and Technology, Clearwater Bay, Kowloon, Hong Kong China; School of Life Sciences, Tsinghua University, Beijing, 100084 China; Current address: Department of Neuroscience, The University of Texas at Southwestern Medical Center, Dallas, TX USA; Department of Electrical, Computer and Biomedical Engineering, University of Pavia, via Ferrata 1, 27100 Pavia, Italy

**Keywords:** Dscam, Homophilic binding, Evolution of arthropods

## Abstract

**Background:**

*Drosophila* Dscam1 is a cell-surface protein that plays important roles in neural development and axon tiling of neurons. It is known that thousands of isoforms bind themselves through specific homophilic interactions, a process which provides the basis for cellular self-recognition. Detailed biochemical studies of specific isoforms strongly suggest that homophilic binding, i.e. the formation of homodimers by identical Dscam1 isomers, is of great importance for the self-avoidance of neurons. Due to experimental limitations, it is currently impossible to measure the homophilic binding affinities for all 19,000 potential isoforms.

**Results:**

Here we reconstructed the DNA sequences of an ancestral Dscam form (which likely existed approximately 40 ~ 50 million years ago) using a comparative genomic approach. On the basis of this sequence, we established a working model to predict the self-binding affinities of all isoforms in both the current and the ancestral genome, using machine-learning methods. Detailed computational analysis was performed to compare the self-binding affinities of all isoforms present in these two genomes. Our results revealed that 1) isoforms containing newly derived variable domains exhibit higher self-binding affinities than those with conserved domains, and 2) current isoforms display higher self-binding affinities than their counterparts in the ancient genome. As thousands of Dscam isoforms are needed for the self-avoidance of the neuron, we propose that an increase in self-binding affinity provides the basis for the successful evolution of the arthropod brain.

**Conclusions:**

Our data presented here provide an excellent model for future experimental studies of the binding behavior of Dscam isoforms. The results of our analysis indicate that evolution favored the rise of novel variable domains thanks to their higher self-binding affinities, rather than selection merely on the basis of simple expansion of isoform diversity, as that this particular selection process would have established the powerful mechanisms required for neuronal self-avoidance. Thus, we reveal here a new molecular mechanism for the successful evolution of arthropod brains.

**Electronic supplementary material:**

The online version of this article (doi:10.1186/s12862-014-0186-z) contains supplementary material, which is available to authorized users.

## Background

For proper axon guidance during neuronal development, it is of critical importance that neurons can distinguish self-connection from non-self connection. for example, neurons must avoid fasciculating or connecting to themselves, in order to spread efficiently into a large area. It has has previously been proposed that specific cell surface molecules provide “labels” for different neurons to distinguish self from non-self, thus allowing the formation of functional neuronal connections [1–6]. Given the diverse types of neurons in the developing nervous system and the number of genes expressed in the brain, the self-recognition of a single neuron is likely to involve multiple genes that encode cell surface molecules. Alternatively, neurons can rely on a large set of diverse molecules derived from a single gene, such as Dscam1 (Down syndrome cell adhesion molecule) in *Drosophila* [1,7].

The Dscam1 gene was shown previously to code for thousands of protein isoforms, namely by way of alternative splicing. Three extracellular Ig domains of Dscam1 (Ig2, Ig3 and Ig7) are encoded by three variable exons (exon4, exon 6 and exon 9), which are very similar in their amino acid sequences [3,4]. Each variable domain is encoded by a corresponding cluster of multiple alternative exons. The numbers of alternative exons in the clusters are 12 (exon 4), 48 (exon 6) and 33 (exon 9) [3,4]. Only one exon from each cluster is used through the stochastic expression process of alternative splicing, to express thouthands of Dscam1 isoform at a time. Therefore, exon combinations from 93 exons (12 + 48 + 33) can form potentially as many as 19,008 (12×48×33) kinds of isoforms with domains with different binding affinity [4,8]. Earlier biochemical *in vitro* studies suggest that each Dscam1 isoform has extracellular domains that prefer to bind to itself (self-binding or homophilic binding), while heterophilic binding (binding between different isoforms) tends to be weak or below the detection limit of the assays used [9]. Importantly, *in vivo* studies with fly mutant forms with reduced Dscam1 expression suggested that the diversity of the Dscam1 isoform population, rather than the specific individual isoforms, is critical for proper neuronal connectivity *in vivo* [1,7], although allele-specific phenotypes raised from few isoforms were observed for a small number of specific Dscam1 mutants [10].

Great diversity of Dscam1 isoforms have been observed across all arthropods [11], and was found to be highly conserved in genomes spanning at least 250 million years of evolutionary distance. Thus, the question arises as to how variable exon usage contributes to the enormous diversity of Dscam1 isoforms in arthropods? Several recent investigations have addressed this question [12–14]. Comparative sequence analysis using fruit fly, mosquito and honeybee revealed that although the sequence diversity of *Dscam1* is high, the underlying mechanisms for generating thousands of isoforms appears to be shared among all three species [15]. The main route to generate these exons was believed to be through reiterative exon duplication and deletion [11,15]. A survey of 16 arthropod as well as non-arthropod genomes further suggested that these tandem exons are limited to arthropods only [16]. The largest study on this topic so far was based on 2000 duplicated exons across 20 arthropods, revealed that the evolutionary occurrence of newly-derived (ND) exons is continuing in the exon 6 and 9 clusters to this day [17]. Together, these reports suggest that Dscam1 diversity plays a critical role in arthropod biology, and more importantly, highlights its unique evolutionary properties comparing to other cell surface molecules and their families.

Although those studies shed light on how such complex gene regulation evolved in arthropods, the precise role of a preference for homophilic binding and its development throughout evolution remains to be elucidated. It is still unclear as to whether the Dscam1 exons were duplicated randomly and retained indiscriminately, until the isoform population reached a threshold level of diversity. ND-exons might then have been further selected based on properties such as the functional or expressional differences of different isoforms. For instance, although Dscam1 is broadly expressed in the nervous systems, there is evidence for R7 cells of the *Drosophila* eye that suggests that not all isoforms are truly randomly expressed, and some isoforms preferably express certain exons of Ig7 [18].

Both *in vitro* and *in vivo* experiments for *Drosophila* Dscam1 suggest that homophilic binding preference is tightly correlated with its functions [1]. In this study, we explored the connection between the evolution of alternative exons on the one hand, and the self-binding affinities of the resulting isoforms on the other. By extracting sequence features of isoforms using available biochemical data, we established a model to predict with high confidence the homophilic binding affinities of these isoforms. Additionally, an ancestor Dscam1 sequence was constructed using genomic information from 12 *Drosophila* species that was approximately 40–50 million year old. Comparative analysis of the ancient and current Dscam1 on their homophilic binding affinities revealed that the current Dscam isoforms have higher homophilic binding affinities than their ancestor. Furthermore, the same conclusion was reached using data from another phylogenetic reconstruction of the exon 6 and 9 clusters [17], verifying the robustness of our analysis. Together, these results highlight a coherent driving force underlying the extensive exon diversification processes of Dscam in *Drosophila* species.

## Results

### The homophilic binding affinity of Dscam1 isoforms can be confidently predicted following sequence analysis

It was shown previously that alternative splicing of the three variable exons (exon 4, 6 and 9) of Dscam1 can generate as many as 19,008 ecto-domain isoforms, and those isoforms assist neuronal cells to distinguish self from non-self through homophilic binding [1,4,9]. Despite such a large number of possible isoforms, the binding affinity between the Dscam1 proteins of the same isoform is particularly high compared to different isofroms [19]. Previous studies on Dscam1 suggested that the observed homophilic binding is mainly determined by the primary protein sequences [19,20]. Thus, it should be possible to analyze the sequence information of all possible isoforms to obtain their homophilic binding affinities relative to heterophilic binding, which would be a time and resource-consuming task if performed using experimental approaches. Estimating the binding affinities of every possible Dscam1 isoform may help to further investigate quantitatively the behavior of neurons expressing combinations of various Dscam1 isoforms.

We identified a total of 89 Dscam1 isoforms from published biochemical studies, for which the self-binding affinities were measured experimentally, and then selected the sequence features that correlated strongly with the homophilic binding affinities of the isoforms (Additional file 1: Table S4). Based on these sequence features and the motif conservation information, the binding affinities of Dscam1 isoforms were predicted using a Support Vector Regression (SVR) model, coupled with feature selection performed via ReliefF Attributes algorithm (RA) (see Additional file 1: Methods and Supplementary Material). To assess our models, we measured the Pearson correlation coefficient (*r*) and Root Mean Square Error (RMSE) from our predictions, which were performed on separate datasets not used to train our models. The correlation coefficient *r* indicates the level of correlation between the binding affinity predicted by our models and the affinity measured by biochemical experiments. The results are shown in Table 1, where *r* and RMSE were obtained as the average of a 10-fold cross-validation. Note that the prediction of binding affinity in our model comes from the combination of sequence features from the entire lg2, lg3 and lg7 domain, rather than individual variable domain, as the synergistic binding of all these domains is likely higher than the sum of the individual binding affinity of lg2-lg2, lg3-lg3 and lg7-lg7.

**Table 1:** **Performance on independent test sets varies with different features**

**Features**	**Pearson correlation coefficient (r)**	**Root mean square error (RMSE)**	**Initial feature size**	**Filtered feature size**
Composition alone	0.61	0.137	40	35
Composition & exon labels	0.65	0.132	43	25
Pseudo amino acid alone	0.74	0.131	120	105
Pseudo amino acid & exon labels	0.65	0.131	123	80
Composition & pseudo amino acid	0.66	0.119	160	55
Exon labels alone	0.40	0.140	3	3
All three types of features combined	0.75	0.115	163	55

Best correlation coefficient (r) and related root mean squared error (RMSE) for datasets based on the 3 types of features alone and their combinations with 10 fold cross-validations. The feature size indicates the number of features obtained by juxtaposing the different descriptors (initial) and the one after RA algorithm (filtered).

The best prediction, *r* = 0.75 and RMSE = 0.115, was achieved with a combination of using pseudo-amino acids, sequence composition and exon labels (exon 4, 6 and 9) constituting 55 encoding features (Additional file 1: Supplementary material and methods). Of these parameters, 43 features were pseudo-amino acids, 11 were composition and 1 was exon label. The ranked attributes and their descriptions are presented in Additional file 1: Table S4.

The Mean Absolute Error (MAE) on test data unknown to the model with our best parameter combination was 0.09 (p < 0.0001, *p* was determined by re-training the model and cross validating datasets with shuffled affinity values). As the affinity values were normalized in the [0, 1] interval, this means the average error was less than 10% on average, indicating that our model can predicate self-binding affinities of Dscam1 isoforms with high confidence on cross-validated data. Therefore, we re-trained the model on all available data, and then applied it again to calculate the self-binding profiles of all Dscam1 isoforms.

### The predicted binding profiles for Dscam1 isoforms are consistent with the experimental data

**Figure 1:**
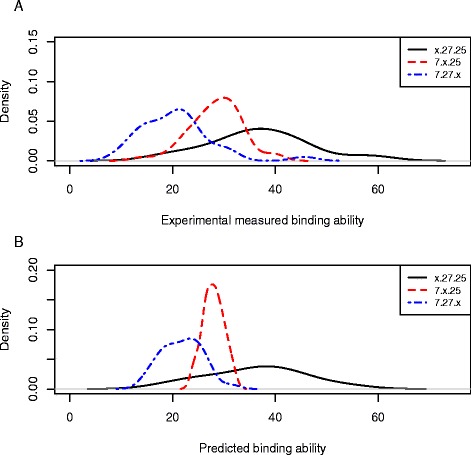
**Profiles of the self-binding affinities of Dscam1isoforms. A**: Binding affinities of the 89 isoforms experimentally measured by Wojtowicz et al. (2007); **B**: Predicted binding affinities for all possible 19,000 isoforms.

The binding profiles predicted by our model were consistent with the values measured experimentally. Firstly, there is a highly significant correlation between the measured binding data and the predicted homophilic binding strength (Pearson’s *r* = 0.86, *p* ≅ 0 and Spearman’s *r* = 0.81, *p* = 3.03e-22, Additional file 2: Table S3). Secondly, biochemical studies chose the isoforms containing exons 4.7, 6.27 and 9.25 as a general example, have shown that the contributions of the variable exons is not equal (Figure 1A), revealing that the isoforms of x.27.25 had the highest average self-binding affinities, while 7.x.25 isoforms showed lower affinities, with 7.27.x exhibiting the lowest self-binding affinities [19]. Our calculation on the Dscam1 isoform population generated a ranking of contributions of the different exons to self-binding affinity: exon 4 > exon 6 > exon 9. Therefore, the model predicted the order of self-binding affinity of the above case as x.27.25 > 7.x.25 > 7.27.x, matching well with previously published biochemical studies [19]. Furthermore, the overall predicted binding affinity for the isoform 7.27.25 (carrying exon 4.7, 6.27 and 9.25) was found to be 28.4, which is in agreement with experimental data published earlier (average value is 30.9 from three experiments) [19]. In addition, the earlier experiments on the variable domain binding specificity based on two different contexts also indicated that the binding domain of exon 4 has the highest binding fold over the assay background, and that exon 9 has the lowest binding affinity [19], which is precisely the order we obtained by prediction (Figure 1B). Furthermore, of the 10 isoforms with the highest self-binding affinities, all contain exon 4.4, and eight contain either exon 9.20 or 9.25. This strongly suggests that the distribution of isoforms with high affinity to self is non-random among the duplicated exons present in the Drosophila genome.

### Reconstruction of the *Drosophila* ancestor dscam1 sequence reveals that isoforms carrying ND-exons have higher self-binding affinities

The complex structure of *Dscam1* gene may have evolved along two possible evolutionary pathways: 1) changing the number of alternative exons by exon duplication and deletion; 2) changing the composition of individual exons by point mutations (including substitution, insertion and deletion). The first path generates ND-exons without clear orthologs, while in the second pathway exons persist throughout evolution (“conserved exons”), preserving identifiable ancient orthologs.

Whilst the evolutionary process of exon duplication is primarily responsible for the generation of the vast number of alternative exons of Dscam1, the actual driving force responsible for this extensive duplication remains unknown [15]. As self*-*binding is an integral part of the control processes involved in cellular recognition during neuronal development, we hypothesized that the Dscam protein evolved in such way in order to improve the homophilic binding affinity of its isoforms. Testing this hypothesis requires a comparison between the current and the ancient forms of the Dscam1 protein. To this end, we first reconstructed a phylogenetic tree for Dscam1, and based on the alignment of the protein sequences of 12 *Drosophila* species, we then rebuilt the ancestral genomic sequence of Dscam1 originating from approximately 40–50 million years ago [21]. We compared the predicted self-binding affinities of different isoforms based on the information derived from both the ancestral and current genomes.

The reconstructed ancestral sequence of Dscam1 contains 62,075 nucleotides (213 bp longer than the current Dscam1 sequence), resulting in a total of 10,920 ancestor isoforms. The GC content of the ancestral and current Dscam1 sequences were found to be similar (45%). However, we found several differences related to their exon variants. Notably, no orthologs were found in the ancestral genome for several current exons, including exon 4 (4.10, 4.11), exon 6 (6.10, 6.20, 6.29, 6.30, 6.40), and exon 9 (9.5, 9.10, 9.13, 9.17, 9.20, 9.24, 9.30). Therefore, those exons would have been generated more recently, probably within the past 40 ~ 50 million years, by exon duplication, while the other exons would already have existed, and would have continued to evolve by point mutations. Here we define “new” isoforms as recently evolved variable domains (through exon duplication) and “ancient” isoforms as those containing conserved domains (evolved by point mutations).

**Figure 2:**
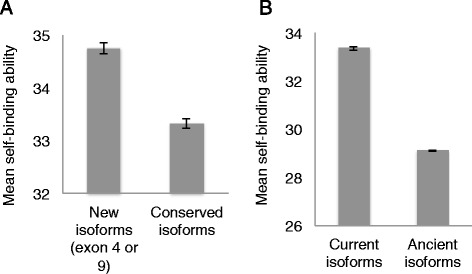
**Comparison of self-binding affinities of isoforms containing current and ancient exons. A**: Distributions of self-binding affinities of isoforms containing exons originating from ancient exon 4 or 9 (conserved isoforms) and those containing exons without such ancient origins (isoforms containing recently acquired exon 4 or 9). Isoforms containing recently gained exons display higher self-binding affinities than conserved isoforms; **B**: Distributions of self-binding affinities of the ancient and current isoforms. The self-binding affinities of current isoforms are higher than those of ancient isoforms.

We set out to investigate as to whether the recently generated isoforms (by exon duplication) display enhanced self-binding affinities relative to the ancient isoforms (Additional file 2: Table S3). We found that all of the isoforms carrying recently duplicated exons have significantly higher predicted self-binding affinities than those with ancient exon orthologs (the conserved isoforms). The mean self-binding affinity is 34.4 ± 0.1 for the new isoforms containing recently appeared exons in exon 4, 6 or 9, with a mean self-binding affinity of 33.4 ± 0.1 for the conserved isoforms (*p* = 8.8e-14, Wilcoxon rank-sum test with continuity correction). Furthermore, the isoforms containing a recently duplicated exon 4 or exon 9 have greater self*-*binding affinities than the isoforms containing corresponding ancient exon 4 or exon 9 (mean binding affinities of new vs. conserved isoforms: exon 4:35.7 ± 0.1 *vs.* 33.4 ± 0.08, *p* < 2.2e-16; exon 9: 34.2 ± 0.1 *vs.* 33.7 ± 0.08, *p* = 0.003, Wilcoxon rank sum test, Figure 2A). Together, these results suggest that recent exon duplications strongly correlate with improved self-binding affinities of the resultant Dscam1 isoforms.

We next tested whether these results could be reproduced by using an independent dataset. The evolutionary history of all of the exons 4, 6 and 9 clusters in 20 arthropods had been independently reconstructed previously [17], and it was reported that nine forms of exon 6 (6.1, 6.30, 6.31, 6.33, 6.34, 6.35, 6.38, 6.47, 6.48) and thirteen of exon 9 (9.1-9.11, 9.32, 9.33) originated approximately 450 million years ago. Using this data set, we found a similar trend to that observed in the dataset described above, namely that all isoforms carrying the recently duplicated exons 6 or 9 display significantly higher self-binding affinities compared to those carrying conserved forms of exon 6 or 9 (mean binding affinities of new vs. conserved isoforms: exon 6: 33.9 ± 0.07 *vs.* 33.5 ± 0.2, *p* = 0.015; exon 9:34.0 ± 0.09 *vs.* 33.5 ± 0.1, *p* = 0.003, Wilcoxon rank sum test). Moreover, the isoforms containing both of the recently duplicated exons 6 and 9 have higher self*-*binding affinities than the isoforms with ancient exons 6 as well as ancient exon 9 (34.1 ± 0.1 for both recent exons, and 33.2 ± 0.2 for both ancient exons, *p* = 0.002, Wilcoxon rank sum test). Taken together, these results indicate that Dscam1 isoforms containing evolutionarily newer exons tend have higher homophilic binding affinities than ancient forms.

### Ancestral dscam proteins display lower self-binding affinities than recent forms

After investigating the self-binding affinities of Dscam isoforms containing exons that have emerged 40–50 million years ago, we analyzed the self-binding profiles of the more conserved isoforms whose exons evolved by point mutations, and for which current-ancestor orthologs exist. The mean self-binding affinities of all ancestor isoforms was shown to be 29.1 ± 0.03, which is significantly lower than that of the current isoforms derived from those ancestor isoforms, which was shown to be 33.4 ± 0.09 (*p* < 2.2e-16 in pair-wise Wilcoxon signed rank test, Figure 2B). These results indicate that the self-binding affinities have increased considerably in this particular isoform population during the last 40 ~ 50 million years of evolution.

**Figure 3:**
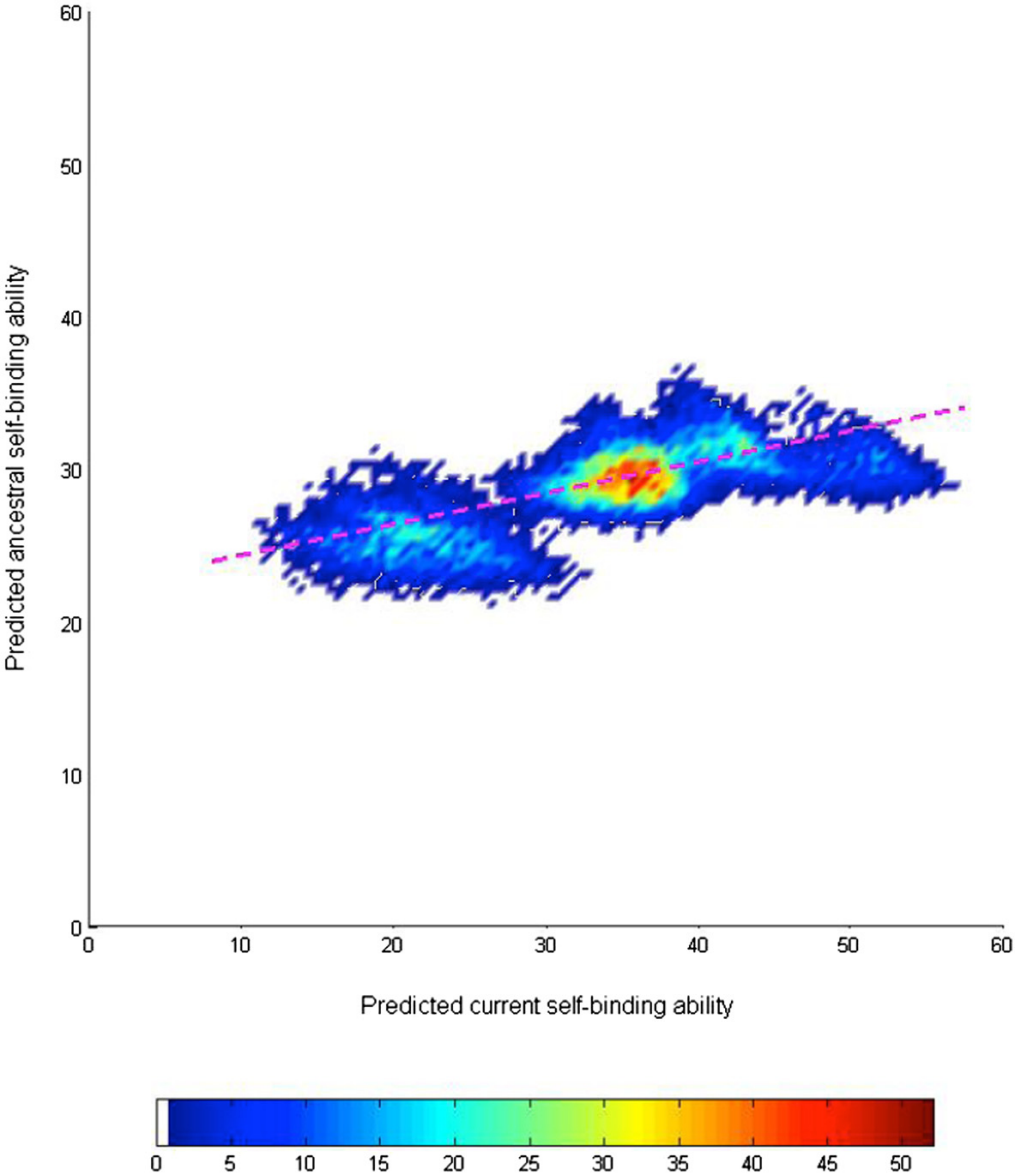
**Correlation of self-binding affinities of current isoforms with those of their ancestral forms.** 10920 Dscam1 isoforms were grouped together: in the figure. The number of isoforms at each data point is presented in pseudo-color as indicated by the color bar below. The dotted purple line indicates a positive correlation of self-binding affinities between time points.

Upon further analysis of each pair of the current-ancestor orthologs it was revealed that there is a strong and significant correlation of self-binding affinities between the ancestor and current isoforms: isoforms with higher binding affinity in the past also have higher self*-*binding affinity at present (*r* = 0.73, *p* ≅ 0, Spearman correlation method, Figure 3). This suggests that any point mutations that might have rendered self-binding to be of lower affinities were either suppressed or eliminated during evolution.

The fact that higher homophilic binding affinity was observed in the current and newly evolved isoforms appears to contradict a neutral evolutionary model of Dscam evolution. If evolving towards a more diverse population were the only selective pressure, and homophilic binding affinity played no role in the evolution of Dscam isoforms, what would happen? A neutral evolutionary model would suggests that, assuming the mean binding affinity of all the isoforms is X in the ancestor Dscam isoforms 40–50 million years ago, the mean binding affinity should be unchanged in both the isoforms containing newly duplicated exons and the conserved isoforms (both are X), and there would be no differences between them and their ancestral isoforms. Our data suggest that this is not the case for Dscam1 evolution. Thus, this neutral evolutionary model is rejected.

## Discussion

**Figure 4:**
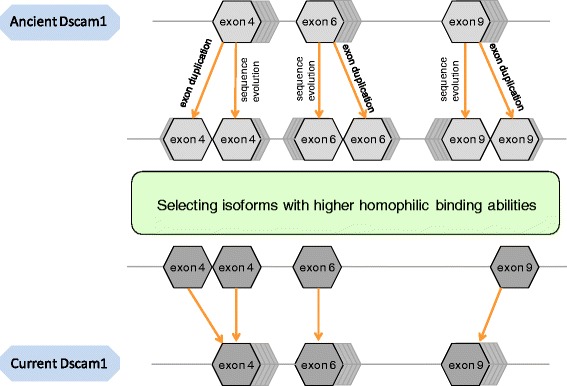
**A schematic model depicting the evolution of Dscam1 in arthropods.** ND-exons were created by exon duplication and/or sequence mutation. Over time, all isoforms containing old and ND-exons were under selection pressures for higher homophilic binding. Only those exons contributing to higher homophilic binding affinities were retained throughout evolution, and can be found in current genome.

Both *in vivo* and *in vitro* studies of Dscam have revealed that highly specific homophilic binding, which may arise from high binding affinities, is a general property of these cell surface proteins, and that this property is crucial for its proper physiological function [1,4,20]. However, it is not feasible to assess experimentally the binding characteristics of all the possible isoforms. Similarily, structure prediction and modeling of all Dscam1 isoforms for which crystal structures are available would be tremendously labor-intensive and time-consuming. Thus, to extract the principle relationship between biochemical binding data and sequence features, we used machine-learning methods to develop a computational model to predict Dscam1 self-binding affinities with high confidence. The self-binding affinities of all isoforms in *Drosophila* genome and those of an ancestral genome, which diverged 40 million years ago, were calculated, and detailed computational comparisons were made to examine a) the binding affinities of the isoforms carrying recently generated exons and ancient exons, and b) the self-binding affinities of those current isoforms that are orthologs of ancient isoforms. Both comparisons revealed that the self-binding affinity of Dscam1 became significantly enhanced over evolutionary time scales, suggesting that the Dscam1 protein evolved to improve the its self-binding affinities, by sequence mutation and/or exon duplication (see Figure 4 for an evolutionary model of Dscam1).

A fascinating feature of Dscam1 is its enormous isoform diversity, which was shown experimentally to be crucial for proper neural development and functioning immune responses in arthropods. Our results presented here suggest that diversity generated simply by exon duplication and sequence mutation may not be adequate, and that a neutral evolutionary model based solely on isoform diversity cannot explain this phenomenon. Even the molecular diversity (large number of isoforms) and biochemical diversity (isoform specific self-binding) together are likely not sufficient. It appears that out of the pool of possible Dscam1 isoforms that existed in the past, the evolutionary process selected only those isoforms that displayed enhanced self-binding affinities (Figure 4). Therefore, diverse isoforms are distinguished from each other not just by composition, but also by their increasingly specialized and progressively enhanced interactions.

We also noticed that the binding affinity we studied here does not equal to binding specificity, and both of them may play important roles in the evolution of Dscam. One may argue that a small improvement in the self-binding affinity would be not sufficient for generating visible benefit in the current Drosophila genome. As the discrimination between self and non-self neurons needs thousands of Dscam isoforms, even a small change in the self-binding affinity of each Dscam isoform would lead to significant differences at the population level of these isoforms, likely causing adequate biological effects as a consequence. Lastly, because of accumulation effects, it would be reasonable to suggest that the 15% increase in self-binding affinity in each isoform when compared to its own ancient isoforms might be crucial for the insect brain to avoid mistakes in the self-recognition process.

Our computational predictions of the self-binding affinities of all possible Dscam1 isoforms demonstrated the potential machine learning methods have for the rapid extraction of key molecular features, as well as for the systematic prediction of biological effects, which is crucial for the accurate large-scale analysis of complex molecular and cellular systems. Because so far, only a small fraction of Dscam1 isoforms has been measured experimentally, the experimental datasets available for our analysis were relatively small. However, the high correlation between measured binding affinities and predicted binding affinities illustrated the power of using such methods of support vector machines on small sample sizes. Profiling the homophilic binding characteristics of just 50% of Dscam1 isoforms would require almost 10,000 experimental measurements, even more if duplications were to be included, a scale that would require facilities for robotic liquid handling of cell transfections as well as automatic binding assays. It needs to be stressed that careful acquisition of experimental data is vital for obtaining accurate and meaningful modeling results. In this context, we recently became aware of a high-throughput domain docking method, which has the potential of simulating self-binding affinities [3]. However, this method requires detailed structural information for each protein, and thus is far more time-consuming when compared to computational modeling. So far, there is no indication as to whether other methods will become available soon for large-scale evolutionary studies like the one discussed here.

It is well-established that homophilic binding of Dscam1 isoforms is crucial for proper functioning in a physiological setting. Therefore, evolutionary selection towards higher affinities towards self is likely to be crucial. Through random processes such as exon duplication and sequence mutation, newly evolved Dscam1 isoforms would have acquired higher self-binding affinities over old isoforms. Selective pressure would then have acted upon these newer Dscam1 isoforms to retain those with higher affinities for self. We speculate that strong self-interactions would provide higher signal-to-noise ratios for cell-cell recognition, which might be just one event among many simultaneous molecular interactions on the cell surface. Such enhanced contrast for meaningful cell-to-cell signaling might ensure that neurons in the developing brain recognize and connect only to appropriate cell partners, in a correct manner. As Dscam1 is also critical during the launch of innate immune responses in insects, self-binding may also play a role in the proper functioning of the immune system.

As one of the most complex molecules in the genome of arthropods, Dscam1 has been studied in great detail using biochemical, genetic and structure modeling approaches. Our comparative profiling of the self-binding affinities of the vast number of Dscam1 isoforms at different time points throughout evolution demonstrates that an evolutionary approach is a powerful and complementary tool to gain novel insights into the mechanistic details of a complex biological system.

## Conclusions

The mechanisms behind the great diversity of arthropod Dscam1 forms containing new variable domains consistently emerging is not just a challenging question in neurobiology, but is equally important for our understanding of evolutionary processes in general. The extensive diversity, in combination with the observed specific homophilic interactions of Dscam1 isoforms provides an unique opportunity for biologists not only to trace the process of its evolution, but also to understand the driving forces behind the evolution of a specific gene at the molecular level. In this study we demonstrated that those Dscam1 isoforms generally evolved to gain higher hemophilic binding affinity during the evolution of arthropod.

## Methods

### Genomic sequences and homophilic binding data of dscam1 proteins

Genomic sequence data and annotations for the Dscam1 protein were obtained from the NCBI gene database. Variable domain sequences were translated into peptide sequences using a self-written Perl script, and manually confirmed by comparing with the literature [4]. All 93 exons (12 exons for variable Ig2 domain, 48 exons for variable Ig3 domain and 33 exons for variable Ig7 domain) were utilized for calculating relevant features of the model (see Feature Encoding). Experimental data for Dscam1 isoforms, consisting of 89 Dscam1 samples of known self-binding affinities, were obtained from previously published reports [19]. To obtain a high-quality model, only data from those experiments that tested variable domain binding specificities were included. For isoforms that were measured multiple times in these experiments, the binding affinity was averaged before use in our model, to prevent biased prediction in our model due to duplication. Although there were some variations in the repeated binding values, due to experimental shortcomings, most of values obtained previously fell into the same range as the affinity values we used for the modeling (9.6-58). All isoforms and corresponding affinities used are listed in the Additional file 1: Table S1.

### Calculation of the phylogenetic relationship of Dscam1 protein and the ancestor sequences

The multiple sequence alignment of *Dscam1 *genes was obtained for 12 *Drosophila* genomes from the UCSC Genome Browser database [22]. The phylogenetic relationship of Dscam1 homologs was calculated using Mrbayes software [23], and the ancestor sequences of 12 *Drosophila* species (*D.melanogaster, D.simulans, D.sechellia, D.yakuba, D.erecta, D.ananassae, D.pseudoobscura, D.persimilis, D.willistoni, D.virilis, D.mojavensis,* and *D.grimsh,* see also http://rana.lbl.gov/drosophila/pseudoobscura.html) were calculated using a maximum-likelihood method within the Ancestors 1.0 software package, using default parameters [24].

### Feature set selection and regression method

We took a regression approach to build a model based on various feature sets. The Support Vector Regression (SVR) method was chosen because of its generalization affinity based on a small number of training samples. Detailed information is provided in the Additional file 1: Table S5.

Briefly, three types of features were extracted: sequence composition, pseudo-amino acids and exon labels. Only the sequences from variable exons (exon 4, 6 and 9) were used, as they provide critical information for the variance of self-binding affinity of Dscam1. In order to identify the best feature combination for our model, the above features were analyzed in a number of combinations.

Composition-based features lead to good correlation (*r* = 0.61 and RMSE =0.137) with 35 features selected, and therefore only 5 of the initial 40 features were ruled out by the ReliefF Attribute (RA) algorithm. Compared to composition features, pseudo-amino acid features appeared to be better suited for describing the Dscam1 self-binding properties: they generated a better correlation (*r* = 0.74 and RMSE = 0.131) with 105 selected features (out of 120). In contrast, the model solely based on exon labels exhibited a lower correlation (*r* = 0.4) and higher RMSE (~0.14).

Combining exon labels with the composition features, we noticed a slight increase in the performance over composition alone: *r* = 0.65 and RMSE = 0.132, with 25 features. Combining exon labels with the pseudo-amino acid features did not improve the general performance of the model (*r* = 0.65), although the number of selected features was fewer (80 out of 123). Combining composition and pseudo amino acid features, we obtained *r* = 0.66 and RMSE = 0.119, and the number of selected features was greatly reduced (55 out of 160). However, the best prediction (*r* = 0.75 and RMSE = 0.115, 55 features) was achieved when all three types of features were combined.

Both composition features and pseudo amino acid features mainly reflect the local properties of a protein. Previous studies indicated that the self-binding of Dscam1 isoform is modular, which means that each of the three variable Igs from one protein binds independently to its counterpart (the identical Ig) on the opposing molecule: Ig2 binds to Ig2, Ig3 to Ig3, and Ig7 to Ig7. This unique binding pattern implicates that the local features within each exon are likely to provide relevant information for self-binding and therefore yield better predictions, while global properties across binding domains, such as exon label features, which describe the higher order organization of different exons, are unlikely to contribute significant information for modular binding. Therefore, our model is inherently consistent with those earlier experimental findings, and was thus able to identify biologically meaningful features.

With the selected features as the input and the affinity of self-binding as output, a ten-fold cross validation procedure was applied to tune the regression parameters so to minimize residual errors. The ReliefF Attribute (RA) algorithm [25] was used to rank the features according to their relevance as calculated by the cross validation. Every train fold had the RA algorithm applied independently, with no influence from its test fold data. Note that this procedure excluded the test sets were from the model training. The parameters for model tuning were the value of γ (the parameter of the RBF kernel) and the number of features. In the final model, those parameter combinations were used that provided the best RMSE on the 10 fold cross-validation. All the tested combinations of γ and filtered feature numbers are listed in the Additional file 1: Table S2. Both RA feature selection and SVR were implemented using the WEKA software package [26].

### Scripts and statistics

The scripts used to process the sequences were written in Perl, and all statistical tests were conducted in R. Wilcoxon rank sum test was used to determine whether two datasets possess significantly different mean values. Pearson or Spearman methods were used to calculation the correlation reported (Specified in the Results section).

## Additional files

Additional file 1: **Supplementary Materials and Methods section and supplementary Tables and Figures.**

Additional file 2: **Supplementary table S3.**
