# Supplementary material for: Improvement of Dscam homophilic binding affinity throughout Drosophila evolution
Source: BMC Evol Biol. 2014 Aug 27;14:186. doi: 10.1186/s12862-014-0186-z (PMC4243935; doi:10.1186/s12862-014-0186-z)
Supplement: Supplementary file 1 — Supplementary Materials and Methods section and supplementary Tables and Figures. [file 12862_2014_186_MOESM1_ESM.doc]

**Supplementary Materials**

**Improvement of Dscam homophilic binding ability throughout Drosophila evolution**

Guang-Zhong Wang (1, #, &), Simone Marini (2, 3, #, $), Xinyun Ma (2), Qiang Yang (3), Xuegong Zhang (2, 4, *), Yan Zhu (1, *)

1. State Key Laboratory of Brain and Cognitive Science, Institute of Biophysics, Chinese Academy of Sciences, 15 Datun Road, Beijing 100101, China

2. Bioinformatics Division, TNLIST/Department of Automation, Tsinghua University, Beijing 100084, China

3. Department of Computer Science and Engineering, Hong Kong University of Science and Technology, Clearwater Bay, Kowloon, Hong Kong, China.

4. School of Life Sciences, Tsinghua University, Beijing 100084, China

# These two authors contributed equally and should be regarded as the joint first authors.

* Corresponding authors.

& Current address: Department of Neuroscience, The University of Texas at Southwestern Medical Center, Dallas, TX, USA.

$ Current address: Laboratory for Biomedical Informatics, via Ferrata 1, Università degli Studi di Pavia, Italy.

.

**Supplementary Methods**

**Feature Encoding**

Our goal was to establish models to quantitatively predict Dscam1 self-binding affinities through a regression approach that was based on extracted features and feature combination. The isoforms and corresponding affinities are listed in the Supplementary Table S1. All of the 89 Dscam1 isoforms with measured binding affinities were used, after normalization to the highest binding affinities for each assay. Only the sequences from variable exons (exon 4, 6 and 9) were used, as they provide the information critical for the variance in self-binding ability of Dscam1. The experimental values of binding affinity ranged from 9.6 to 58, and they were normalized to be within the interval [0, 1]. The homophilic binding of isoform 7.27.25 was measured experimentally three times, thus the average value was used here.

We extracted three types of features: sequence composition, pseudo amino acids and exon labels. For sequence compositional features, the frequencies of single amino acids, single nucleotides and dinucleotides were calculated. This approach extracted 40 features for each protein (20 amino acids, 4 nucleotides and 16 dinucleotides).

We also utilized pseudo-amino acid features as inputs for our models, as protein primary structure is often utilized to predict protein-protein interactions. In fact, the knowledge of the amino acid sequence alone might theoretically be sufficient to estimate protein qualities and behavior. However, primary sequences of proteins vary greatly in length, so they could not be used directly, since regression and classification algorithms require a fixed number of features for each instance. This information for predicting the binding affinity would be wrapped, for example, in the compositional features mentioned above. Representing primary sequences with a fixed feature number will lose the positional information. To partially overcome this loss, we proposed the use of pseudo-amino acids instead . We describe protein sequences with a fixed number of features and embed sequential characteristics based on both composition and physicochemical amino acid properties, for example hydrophobicity, which is among the fundamental factors that determine protein folding. Pseudo-amino acids are widely exploited in bioinformatics . To our knowledge, this is the first time that they are applied for quantitative prediction (through regression) of protein interactions. The feature number for the pseudo-amino acids is 20+λ, where λ is a user-defined number. For Dscam1 pseudo-amino acid feature encoding, we set λ=20 to collect 40 pseudo-amino acids features for each alternative exon (20 based on the frequency of the native amino acids, 20 based on hydrophobicity, weighting factor = 0.05), which leads to 40x3=120 features for each isoform.

For the exon label features, a combination of three ordinal labels (one for each of exon 4, exon 6 and exon 9) was used to represent the exon composition of an isoform. Here, each label was utilized as an ordinal feature, not as a number. The label combination uniquely defines a single sample. For instance, for the sample composed of exons 4.10, 6.12 and 9.30, the combination [10, 12, 30] is its exon label feature set. The labels utilized are listed in Supplementary Table S1. The summary of the features extracted for each approach is shown in Supplementary Table S2. To avoid introducing false quantitative relationships between exon labels, an ordinal feature is encoded as multiple binary features. For example, the ordinal feature encoding the twelve labels of exon 4 (4.1, …, 4.12) is defined as twelve binary features. For each particular isoform, only one of these twelve features is “1”, indicating the particular exon being used in this isoform, and all the others are “0”.

**Feature Combinations and Regression Method**

In order to identify the best feature combination to build our model, we combined the three types of features described above in a number of different ways. We first built three datasets with each of the three types of features, and then assembled another three datasets by combining different types of features: one consisting of the composition features and exon label features, one consisting of the pseudo amino acid features and exon label features, and one containing all three types of features. Thus, in total we generated six datasets.

The features compose the input vector of the regression model, the output of which is the affinity of self-binding. We adopted the Support Vector Regression (SVR) method for the regression. SVR is a method that aims at minimizing the residual error and maximizing the generalization ability based on a small number of training samples. Non-linearity is achieved by mapping the original feature space into a high-dimensional space with the RBF kernel. After the prediction, the output is re-scaled from the range of [0, 1] back to the natural range of affinity values.

**Best Feature Set Selection**

Ten-fold cross validation was applied to tune the regression parameters as described below. A dataset was randomly divided into ten subsets of approximately equal size. Nine of the subsets were used to compose the training set and the remaining subset was used as the test set. This produced a total of 10 compositions of training and test sets. The ReliefF Attribute (RA) algorithm was used to rank the features according to the relevance of each feature from the cross validation, taking into account feature interrelationships.

The RA method is based on how well attributes distinguish between similar instances. Given a data set *D* composed by (*D_0_* … *D_i_* … *D_N_*) instances, each one encoded by *n* attributes, RA calculates the distance between an instance pair (*D_x_*, *D_y_*) in the *n-*dimensional space. After obtaining the distance between every possible pair (*D_x_*, *D_y_*) of instances present in the data set, the closest *P* instances to an instance were considered as neighbors of that instance, where *P* is a user defined number. In this way, every instance *D_i_* has an associated neighbor group (*D_n0_*, *D_n1_*, …, *D_nP_*). The predictive importance of each attribute is based on how the attribute value differs among a selected instance and its neighbors, in the following way: starting from a random instance, RA searches among its *P* neighbors and estimates the importance of each attribute, i.e. how a single attribute differs among the instance and the neighbors. Proceeding iteratively, the estimation is updated until all the n instances of the data set have been selected once and their attributes compared to their neighbors.

In our case, we utilized the normalized linear distance and 20 neighbors for each step of the procedure. The parameters for model tuning were the value of γ (the parameter of RBF kernel) and the number of features. For each parameter combination, we created 10 training models (one for each of the 10-folds) and tested them on their respective test sets. Note that with this procedure, the test sets were not involved in model training. Every model was applied to its corresponding test set, and the Pearson correlation coefficient (*r*), Root Mean Square Error (RMSE) and Mean Absolute Error (MAE) of the predicted values and the true values were calculated: RMSE =
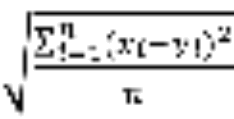
, *r =*
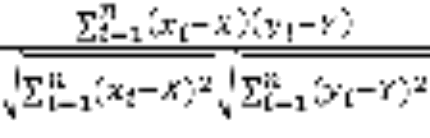
, MAE =
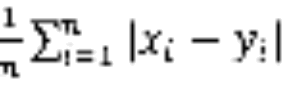
), where *x_i_* is a predicted affinity, *y_i_* is the corresponding measured one,
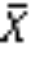
is the average value of the predicted affinities and
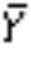
 is the average value of the measured affinities.

The RMSE and its associated *r* for a particular parameter combination (γ and feature number) were the mean of the results over the ten test sets. All the tested combinations of γ and filtered feature numbers are in the Supplementary Table S2. Both RA feature selection and SVR were implemented with the WEKA software.

**Supplementary Tables**

**Table S1**. Sequence features correlated with isoform self-binding abilities.

The frequency of each factor was used in the calculation. The Spearman method was used for calculating the correlation coefficients.

| **Factor name** |  | **Correlation coefficient *r*** | **Significant level *p*** |
| --- | --- | --- | --- |
| D |  | 0.329 | 0.0014 |
| E |  | -0.397 | 9.6e-05 |
| K |  | 0.326 | 0.0016 |
| R |  | -0.209 | 0.047 |
| Y |  | 0.273 | 0.0089 |
| A |  | -0.224 | 0.033 |
| L |  | -0.389 | 0.00014 |
| P |  | 0.213 | 0.043 |
| An |  | 0.228 | 0.030 |
| Cn |  | -0.247 | 0.019 |
| AC |  | 0.247 | 0.018 |
| CA |  | -0.334 | 0.0011 |
| GA |  | -0.217 | 0.038 |
| TA |  | 0.441 | 1.19e-05 |
| TC |  | -0.310 | 0.0027 |
| GT |  | 0.258 | 0.014 |
| TG |  | -0.241 | 0.022 |

Table S2. Listed are the variables that are utilized in the final regression model. Each instance is composed of the 3 protein sequences that correspond to the three variable domains Ig2, Ig3 and Ig7, and their related nucleotide sequences.

Exon label: a label corresponding to the considered instance’s variable domain;

Nucleotide and dinucleotide composition: Normalized frequency of a nucleotide (or a dinucleotide) in the sum of the three nucleotide sequences coding for Ig2, Ig3 and Ig7;

Amino Acid composition: Normalized frequency of AA in the three variable domain;

Pseudo-Amino Acid, frequency-related: Amino acid composition calculated as reported (Chou, 2000);

Pseudo Amino Acid, sequence-related: term that embeds a hydrophobicity pattern present in the sequence of a single domain, considering AAs at a fixed distance (see Chou, 2000).

| **Rank** | **Name** | **Type** | **Feature encoding** | **Description** |
| --- | --- | --- | --- | --- |
| 1 | Exon4 | Ordinal | Exon label | Label identifying each possible instance of variable domain Ig2 (4.1, …, 4.12) |
| 2 | Mex4 | Numeric | Pseudo amino acid | Frequency information of M amino acid in Ig2 |
| 3 | Qex4 | Numeric | Pseudo amino acid | Frequency information of Q amino acid in Ig2 |
| 4 | 32ex4 | Numeric | Pseudo amino acid | Second tier hydrophobicity sequence order-related term in Ig2 |
| 5 | 31ex4 | Numeric | Pseudo amino acid | Second tier hydrophobicity sequence order-related term in Ig2 |
| 6 | Dex4 | Numeric | Pseudo amino acid | Frequency information of D amino acid in Ig2 |
| 7 | Gex4 | Numeric | Pseudo amino acid | Frequency information of G amino acid in Ig2 |
| 8 | Qc | Numeric | Composition | Frequency information of C |
| 9 | 36ex4 | Numeric | Pseudo amino acid | Second tier hydrophobicity sequence order-related term in Ig2 |
| 10 | 29ex4 | Numeric | Pseudo amino acid | Second tier hydrophobicity sequence order-related term in Ig2 |
| 11 | 30ex4 | Numeric | Pseudo amino acid | Second tier hydrophobicity sequence order-related term in Ig2 |
| 12 | 20ex4 | Numeric | Pseudo amino acid | Second tier hydrophobicity sequence order-related term in Ig2 |
| 13 | Ac | Numeric | Composition | Frequency information of A |
| 14 | Yc | Numeric | Composition | Frequency information of Y |
| 15 | Ic | Numeric | Composition | Frequency information of I |
| 13 | 35ex4 | Numeric | Pseudo amino acid | Second tier hydrophobicity sequence order-related term in Ig2 |
| 14 | 38ex4 | Numeric | Pseudo amino acid | Second tier hydrophobicity sequence order-related term in Ig2 |
| 15 | 22ex4 | Numeric | Pseudo amino acid | Second tier hydrophobicity sequence order-related term in Ig2 |
| 16 | Nex4 | Numeric | Pseudo amino acid | Frequency information of N amino acid in Ig2 |
| 17 | Lex4 | Numeric | Pseudo amino acid | Frequency information of L amino acid in Ig2 |
| 19 | 34ex4 | Numeric | Pseudo amino acid | Second tier hydrophobicity sequence order-related term in Ig2 |
| 20 | 37ex4 | Numeric | Pseudo amino acid | Second tier hydrophobicity sequence order-related term in Ig2 |
| 21 | Rex4 | Numeric | Pseudo amino acid | Frequency information of R amino acid in Ig2 |
| 22 | 24ex4 | Numeric | Pseudo amino acid | Second tier hydrophobicity sequence order-related term in Ig2 |
| 23 | Hex4 | Numeric | Pseudo amino acid | Frequency information of H amino acid in Ig2 |
| 24 | 27ex4 | Numeric | Pseudo amino acid | Second tier hydrophobicity sequence order-related term in Ig2 |
| 25 | 27ex4 | Numeric | Pseudo amino acid | Second tier hydrophobicity sequence order-related term in Ig2 |
| 26 | 23ex4 | Numeric | Pseudo amino acid | Second tier hydrophobicity sequence order-related term in Ig2 |
| 27 | Dc | Numeric | Composition | Frequency information of D |
| 28 | 33ex4 | Numeric | Pseudo amino acid | Second tier hydrophobicity sequence order-related term in Ig2 |
| 29 | Fex4 | Numeric | Pseudo amino acid | Frequency information of F amino acid in Ig2 |
| 30 | 26ex4 | Numeric | Pseudo amino acid | Second tier hydrophobicity sequence order-related term in Ig2 |
| 31 | 28ex4 | Numeric | Pseudo amino acid | Second tier hydrophobicity sequence order-related term in Ig2 |
| 32 | Lc | Numeric | Composition | Frequency information of L |
| 33 | Tex4 | Numeric | Pseudo amino acid | Frequency information of T amino acid in Ig2 |
| 34 | Nc | Numeric | Composition | Frequency information of N |
| 35 | 25ex4 | Numeric | Pseudo amino acid | Second tier hydrophobicity sequence order-related term in Ig2 |
| 36 | Eex4 | Numeric | Pseudo amino acid | Frequency information of E amino acid in Ig2 |
| 37 | CTc | Numeric | Composition | Frequency of the dinucleotides CT |
| 38 | TCc | Numeric | Composition | Frequency of the dinucleotides TC |
| 39 | 21ex4 | Numeric | Pseudo amino acid | Second tier hydrophobicity sequence order-related term in Ig2 |
| 40 | Kex4 | Numeric | Pseudo amino acid | Frequency information of K amino acid in Ig2 |
| 41 | Vex4 | Numeric | Pseudo amino acid | Frequency information of V amino acid in Ig2 |
| 42 | Dex4 | Numeric | Pseudo amino acid | Frequency information of D amino acid in Ig2 |
| 43 | Pex4 | Numeric | Pseudo amino acid | Frequency information of P amino acid in Ig2 |
| 44 | Vc | Numeric | Composition | Frequency information of V amino acid |
| 45 | Gc | Numeric | Composition | Frequency information of G |
| 46 | CCc | Numeric | Composition | Frequency of the dinucleotides CC |
| 47 | 107ex9 | Numeric | Pseudo amino acid | Second tier hydrophobicity sequence order-related term in Ig7 |
| 48 | 101ex9 | Numeric | Pseudo amino acid | Second tier hydrophobicity sequence order-related term in Ig7 |
| 49 | Nex9 | Numeric | Pseudo amino acid | Frequency information of N amino acid in Ig7 |
| 50 | 39ex4 | Numeric | Pseudo amino acid | Second tier hydrophobicity sequence order-related term in Ig2 |
| 51 | Hc | Numeric | Composition | Frequency information of H |
| 52 | 102ex9 | Numeric | Pseudo amino acid | Second tier hydrophobicity sequence order-related term in Ig7 |
| 53 | Ec | Numeric | Composition | Frequency information of C |
| 54 | Sex4 | Numeric | Pseudo amino acid | Frequency information of amino acid S in Ig2 |
| 55 | 109ex9 | Numeric | Pseudo amino acid | Second tier hydrophobicity sequence order-related term in Ig7 |

**Table S4.** Self-binding affinities used for model trainings.

The original measured data from Wojtowicz et al. (2007) were corrected for background noise to eliminate variations introduced by experimental procedures.

| **Exon 4** | **Exon 6** | **Exon 9** | **Self-binding affinity after correction for the background** | **Exon 4** | **Exon 6** | **Exon 9** | **Self-binding affinity after correction for the background** | **Exon 4** | **Exon 6** | **Exon 9** | **Self-binding affinity after correction for the background** |
| --- | --- | --- | --- | --- | --- | --- | --- | --- | --- | --- | --- |
| 1 | 27 | 25 | 43 | 7 | 29 | 25 | 32.86 | 7 | 27 | 9 | 21.6 |
| 10 | 27 | 25 | 45 | 7 | 14 | 25 | 25.44 | 7 | 27 | 8 | 9.6 |
| 2 | 27 | 25 | 36 | 7 | 13 | 25 | 33.92 | 7 | 27 | 14 | 14.4 |
| 3 | 27 | 25 | 44 | 7 | 8 | 25 | 32.86 | 7 | 27 | 15 | 14.4 |
| 4 | 27 | 25 | 58 | 7 | 12 | 25 | 29.68 | 7 | 27 | 16 | 13.2 |
| 5 | 27 | 25 | 37 | 7 | 25 | 25 | 26.5 | 7 | 27 | 27 | 24 |
| 6 | 27 | 25 | 25 | 7 | 26 | 25 | 27.56 | 7 | 27 | 17 | 18 |
| 7 | 27 | 25 | 30.9 | 7 | 47 | 25 | 29.68 | 7 | 27 | 24 | 21.6 |
| 7 | 1 | 25 | 33.92 | 7 | 48 | 25 | 20.14 | 7 | 27 | 18 | 21.6 |
| 7 | 2 | 25 | 39.22 | 7 | 7 | 25 | 36.04 | 7 | 27 | 23 | 30 |
| 7 | 3 | 25 | 22.26 | 7 | 6 | 25 | 30.74 | 7 | 27 | 22 | 26.4 |
| 7 | 28 | 25 | 18.02 | 7 | 15 | 25 | 27.56 | 7 | 27 | 19 | 24 |
| 7 | 3 | 25 | 27.56 | 7 | 16 | 25 | 23.32 | 7 | 27 | 2 | 19.2 |
| 7 | 33 | 25 | 26.5 | 7 | 23 | 25 | 23.32 | 7 | 27 | 21 | 22.8 |
| 7 | 35 | 25 | 27.56 | 7 | 22 | 25 | 24.38 | 7 | 27 | 26 | 25.2 |
| 7 | 44 | 25 | 40.28 | 7 | 24 | 25 | 26.5 | 7 | 27 | 28 | 13.2 |
| 7 | 36 | 25 | 31.8 | 7 | 9 | 25 | 29.68 | 7 | 27 | 1 | 10.8 |
| 7 | 4 | 25 | 31.8 | 7 | 19 | 25 | 13.78 | 7 | 27 | 2 | 20.4 |
| 7 | 46 | 25 | 32.86 | 7 | 2 | 25 | 25.44 | 7 | 27 | 3 | 22.8 |
| 7 | 45 | 25 | 29.68 | 7 | 21 | 25 | 22.26 | 7 | 27 | 4 | 20.4 |
| 7 | 41 | 25 | 28.62 | 7 | 18 | 25 | 31.8 | 7 | 27 | 12 | 21.6 |
| 7 | 42 | 25 | 27.56 | 7 | 17 | 25 | 30.74 | 7 | 27 | 32 | 15.6 |
| 7 | 43 | 25 | 32.86 | 7 | 1 | 25 | 26.5 | 7 | 27 | 13 | 45.6 |
| 7 | 37 | 25 | 23.32 | 7 | 4 | 25 | 32.86 | 7 | 27 | 29 | 13.2 |
| 7 | 38 | 25 | 29.68 | 7 | 27 | 31 | 15.6 | 7 | 27 | 3 | 20.4 |
| 7 | 39 | 25 | 25.44 | 7 | 27 | 5 | 22.8 | 8 | 27 | 25 | 36 |
| 7 | 34 | 25 | 21.2 | 7 | 27 | 1 | 18 | 9 | 27 | 25 | 19 |
| 7 | 31 | 25 | 28.62 | 7 | 27 | 11 | 18 | 11 | 27 | 25 | 31 |
| 7 | 32 | 25 | 30.74 | 7 | 27 | 6 | 30 | 12 | 27 | 25 | 38 |
| 7 | 3 | 25 | 29.68 | 7 | 27 | 7 | 15.6 |  |  |  |  |

**Table S5:** Feature selection and γ values.

For each data set, multiple **γ** values (5, 1, 0.5, 0.1. 0.05, 0.01, 0.005, and 0.001) were tested. Each γ was paired with a number of selected features, and ranked using the RA algorithm according to the size of the initial data set.

| **Data set name** | **# of initial features** | **Feature type** | | **# of features considered for selection** | |
| --- | --- | --- | --- | --- | --- |
| Exon Labels | 3 | | ordinal | | None |
| Composition | 40 | | numeric | | 20, 25, 30, 35 |
| Composition and Exon Labels | 43 | | ordinal and numeric | | 20, 25, 30, 35 |
| Pseudo Amino Acid | 120 | | numeric | | 20, 25, 30, 35, 40, 45, 50, 55, 60, 65, 70, 75, 80, 85, 90, 95, 100, 105, 110, 115 |
| Pseudo Amino Acid and Exon Labels | 123 | | ordinal and numeric | | 20, 25, 30, 35, 40, 45, 50, 55, 60, 65, 70, 75, 80, 85, 90, 95, 100, 105, 110, 115 |
| Pseudo Amino Acid and Composition | 160 | | numeric | | 20, 25, 30, 35, 40, 45, 50, 55, 60, 65, 70, 75, 80, 85, 90, 95, 100, 105, 110, 115, 120, 125, 130, 135, 140, 145, 150 |
| Pseudo Amino Acid, Composition and Exon Labels | 163 | | ordinal and numeric | | 20, 25, 30, 35, 40, 45, 50, 55, 60, 65, 70, 75, 80, 85, 90, 95, 100, 105, 110, 115, 120, 125, 130, 135, 140, 145, 150 |

**Reference**:

1. Chou KC: **Prediction of protein subcellular locations by incorporating quasi-sequence-order effect**. *Biochemical and biophysical research communications* 2000, **278**(2):477-483.

2. Robnik-Šikonja, Kononenko I: **Theoretical and Empirical Analysis of ReliefF and RReliefF**. *Mach Learn* 2003, **53**(1-2):23-69.

3. Hall. M, Frank. E, Holmes. G, Pfahringer. B, Reutemann. P, Witten. IHW: **The WEKA data mining software: an update**. *SIGKDD Explor Newsl* 2009, **11**(1):1931-0145.
